# Fulminant central nervous system varicella-zoster virus infection unexpectedly diagnosed by metagenomic next-generation sequencing in an HIV-infected patient: a case report

**DOI:** 10.1186/s12879-020-4872-8

**Published:** 2020-02-19

**Authors:** Mingxia Fang, Xing Weng, Liyun Chen, Yaling Chen, Yun Chi, Wei Chen, Zhiliang Hu

**Affiliations:** 1 Department of Infectious Disease, the Second Hospital of Nanjing, Nanjing University of Chinese Medicine, 1-1 Zhongfu Road, Nanjing, 210003 China; 2 Department of pathogen detection products, BGI-Shenzhen, Shenzhen, 518083 China; 3 Department of Infectious Disease, The Third People’s Hospital of Wuhu, Wuhu, 241000 China; 4 Department of Clinical Research Center, the Second Hospital of Nanjing, Nanjing University of Chinese Medicine, 1-1 Zhongfu Road, Nanjing, 210003 China; 5 Center for Global Health, School of Public Health, Nanjing Medical University, Nanjing, 211166 China

**Keywords:** Varicella-zoster virus, Central nervous system infection, Metagenomic next-generation sequencing

## Abstract

**Background:**

Varicella-zoster virus (VZV) infection can be diagnosed clinically once classical rash occurs but the diagnosis is challenging when typical rash is absent. We reported a case of fulminant central nervous system (CNS) VZV infection in a human immunodeficiency virus (HIV)-infected patient without typical VZV-related rash. CNS VZV infection was unexpected identified by metagenomic next-generation sequencing (mNGS).

**Case presentation:**

A 28-year-old HIV-infected patient presented with neurological symptoms for 3 days. The patient, who was not suspected of VZV infection at admission, quickly progressed to deep coma during the first 24 h of hospitalization. An unbiased mNGS was performed on DNA extract from 300 μL cerebrospinal fluid (CSF) with the BGISEQ-50 platform. The sequencing detection identified 97,248 (out of 38,561,967) sequence reads uniquely aligned to the VZV genome, and these reads covered a high percentage (99.91%) of the VZV. Presence of VZV DNA in CSF was further verified by VZV-specific polymerase chain reaction and Sanger sequencing. Altogether, those results confirmed CNS VZV infection.

**Conclusions:**

This study suggests that mNGS may be a useful diagnostic tool for CNS VZV infection. As mNGS could identify all pathogens directly from CSF sample in a single run, it has the promise of strengthening our ability to diagnose CNS infections in HIV-infected patients.

## Background

Primary varicella-zoster virus (VZV) infection causes varicella (chickenpox), after which VZV becomes latent in ganglionic neurons. In the case of impaired immunity, virus reactivation leads to zoster (shingles), which manifests as unilateral dermatomal rash [1, 2]. VZV infection can be diagnosed clinically once typical rash occurs but the diagnosis is a challenge when classical rash is absent. Here, we reported a case of fulminant central nervous system (CNS) VZV infection in a human immunodeficiency virus (HIV)-infected patient without typical VZV-related skin rash. A Metagenomic next-generation sequencing (mNGS), which acted in a target-independent manner [3], enabled identification of the etiological agent in cerebrospinal fluid (CSF) when we were unaware of VZV infection.

## Case presentation

**Fig. 1:**
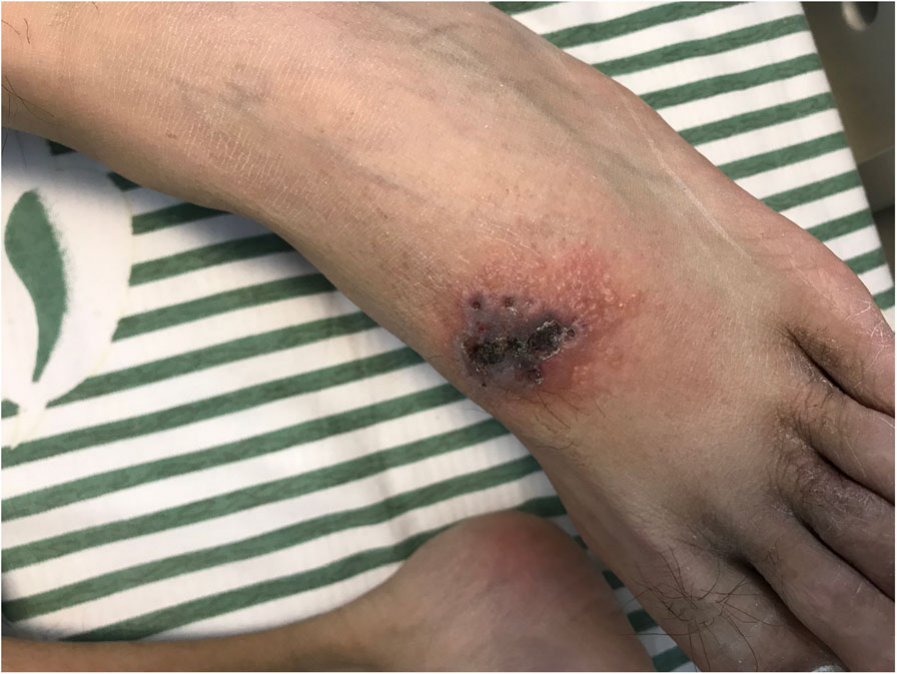
Skin lesion on patient’s left foot. There was a superficial ulcer on the dorsum of the left foot with partial necrosis and black crust, surrounded by a cluster of blisters

A 28-year-old Chinese male was admitted to our hospital because of headache, nausea and vomiting for 3 days. He was confirmed HIV and hepatitis B virus (HBV) co-infection 2 years ago and did not receive anti-viral therapy. In the preceding month, he had been hospitalized in another hospital due to pneumocystis pneumonia that was resolved with cotrimoxazole and corticosteroids. One week before his admission to our hospital, a skin rash developed on his left foot which did not receive much attention. At the time of his admission to our hospital, this rash appears as superficial ulcer with partial necrosis and black crust surrounded by a cluster of blisters (Fig. 1), which was not thought to be linked with VZV infection. The patient was alert with a stiff neck. An emergent brain computer tomography was unremarkable. Twelve hours after admission, the patient complained of difficult urination and had two seizure onsets in the next 12 hours. He lost consciousness after the first seizure onset and lost autonomous respiration after the second seizure onset. A ventilator was then used to control his respiration, and noradrenaline was needed to maintain adequate blood pressure. Combinatory therapy with ceftriaxone tazobactam, linezolid, voriconazole and ganciclovir was administered to ensure the coverage of common bacterial meningitis, cytomegalovirus (CMV) meningitis, as well as CNS fungal infections.

After his admission to our hospital, the main laboratory tests from blood samples were as follows: (1) white blood cell count: 9.41 × 10^9^, N 0.67; (2) plasma procalcitonin<0.1 ng/mL; C reaction protein: (3) negative serum cryptococcal antigen test; negative serum *Treponema pallidum* particle agglutination assay; negative serum tuberculosis antibodies; negative interferon-γ release assay; negative blood culture;(4) plasma CMV load: serum HBV load: 2.18 × 10^8^ copies/mL; (5) CD4 cell count: 137 cells/uL; plasma HIV load: 1.2 × 10^6^ copies/mL. His clinical condition was not improved with the above mentioned combinatory anti-infective therapy. On the third day of the hospitalization, a lumbar puncture was performed. The CSF opening pressure was 15 cm of H_2_O. CSF analysis demonstrated a white blood cell count of 91 cells/ mm^3^, glucose level of 3.28 mmol/L, total protein level of 9.6 g/L, negative India ink staining, negative cryptococcal antigen test, negative gene Xpert MTB/RIF assay and negative culture result. Also, CSF sample was sent for mNGS analysis. The patient was deceased on the fifth day of the hospitalization. The next day, mNGS analysis was completed which revealed high burden of VZV in the CSF. A follow-up telephone visit of the patient’s family member revealed that the patient had a history of chickenpox in childhood. VZV DNA was detected from stored serum sample.

## mNGS

**Fig. 2:**
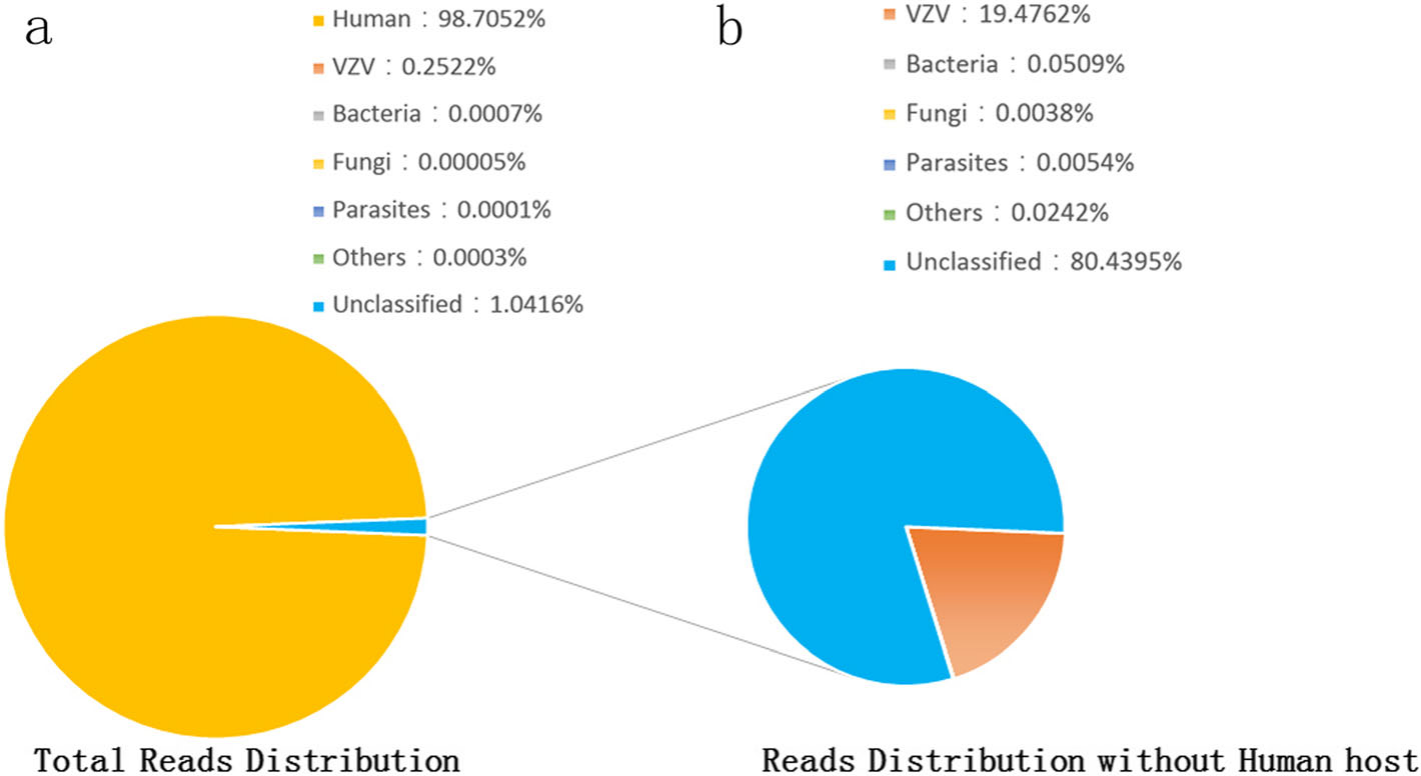
Analysis of sequencing result of varicella-zoster virus using the metagenomic next-generation sequencing. **a** Reads distribution of total DNA in the cerebrospinal fluid samples. **b** Reads distribution of all non-human reads

mNGS was performed in BGI-Shenzhen exactly following our previous protocol [4], including isolation of total DNA, construction of DNA libraries and sequencing. With subtraction of human host sequences [5], the remaining data were classified to four microbial genome databases, containing viruses, bacteria, fungi and parasites associated with human infection. In the meanwhile, a CSF sample was collected from a non-infected patient and applied to the same mNGS procedure. In total, 97,248 sequence reads out of 38,561,967 were uniquely aligned to the VZV genome (Fig. 2), and these reads covered almost the whole VZV genome (99.91%). Strikingly, VZV reads were standing out in all viral species, accounting for 99.91% of total viral reads. Trace HBV and CMV sequences were detected in the CSF sample (62 and 25 sequence reads, respectively). As expect, no VZV read was detected from control sample.

## PCR verification

**Fig. 3:**
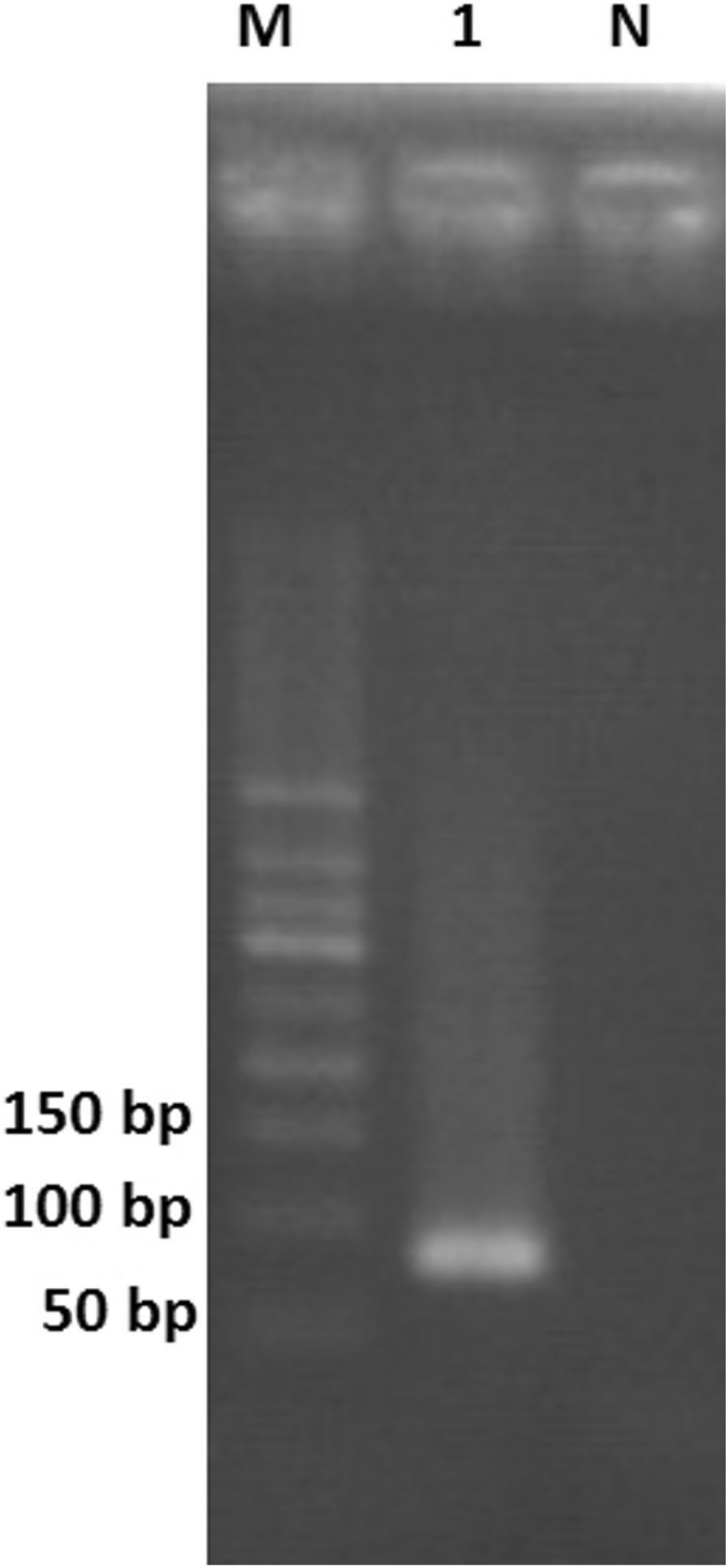
Polymerase chain reaction detection of varicella-zoster virus. Lane 1: 89-bp polymerase chain reaction product of varicella-zoster virus; Lane M: DNA ladder; Lane N: Negative control

To verify the results of mNGS, PCR was performed to amplify the *Pst*I site located within gene 38 of VZV, with VZV-specific primers VZV-F (AAGTTCCCCCCGTTCGC) and VZV-R (TGGACTTGAAGATGAACTTAATGAAGC) [6]. The 89-bp target fragment (Fig. 3) was sequenced and mapped to a reference VZV sequence (GenBank accession no. KP771925.1), with 93% identity. Consequently, all these results indicated that the patient was infected with VZV.

## Discussion and conclusion

To etiologically diagnose CNS infection, conventional methods generally need massive volume of CSF sample to perform a battery of tests, which include smear, culture, nucleic acid amplification assays and serological tests. It is not possible to test all the CNS pathogens using those microbiologic methodologies due to limited amount of CSF sample and commercial test kits available. Recently developed mNGS has the capability to overcome limitations of traditional diagnostic tests. This new technology could identify all pathogens directly from CSF sample with a single run in a hypothesis-free and culture-independent manner [3]. Studies have shown that mNGS is more sensitive than traditional culture method in clinical conditions such as blood stream and respiratory infections [7, 8]. More importantly, due to unbiased sampling, mNGS is theoretically able to identify not only known but also unexpected pathogens or even discovery novel organisms [9]. A recent study showed that mNGS of CSF could improve diagnosis of neurologic infections [10]. Nevertheless, to which extend this strategy could improve our ability of microbiological diagnosing of CNS infection in HIV-infected patients is less clear.

CNS infections are closely associated with morbidity and mortality in HIV-infected patients [11]. Common forms of CNS diseases in HIV-infected patients include cryptococcal meningitis, tuberculosis meningitis, cerebral toxoplasmosis, cytomegalovirus encephalitis, progressive multifocal leukoencephalopathy and HIV-encephalopathy [12, 13]. One of these CNS diseases may be considered as initial diagnosis when an HIV-infected patient presents with neurological symptoms. Usually, clinicians may choose a series of pathogen-specific parameters to confirm the initial diagnosis and make a differential diagnosis. Those conventional diagnostic methods require a prior knowledge of the causative pathogens. When the patient is infected with rare pathogen or has unusual presentation, achieving a timely diagnosis may be difficult. VZV was not thought to be a common cause of CNS infection in HIV-infected patients [13]. As our patient did not have typical VZV related rash, VZV-specific tests were not performed. If mNGS was not applied to this patient, CNS VZV infection would remain undiagnosed. As he had a history of chickenpox in childhood, the latest CNS infection reflected a VZV reactivation. The rash on his left feet may be an ignored atypical presentation of zoster. Of note, VZV encephalitis without rash or radicular pain had been reported. Lacking any clue of VZV infection, the causative pathogen in that case could only be known after a brain biopsy [14]. In the future, it was possible that more atypical cases of CNS VZV infection could be identified using mNGS on CSF sample.

The clinical practice in our patient suggests that mNGS has the promise of strengthening our ability to diagnose CNS infections in HIV-infected patients. Firstly, HIV-infected patients could suffer from infections that are not commonly seen in general populations. The commercial kits for testing those relatively rare pathogens are often unavailable in many regions. Secondly, HIV-infected patients may have atypical presentations of common pathogens. For example, zoster that due to reactivation of VZV infection is a common clinical condition in HIV-infected patients. However, due to unaware of the atypical infection, CNS VZV infection was not known and not interrogated by traditional methods until mNGS demonstrated the presence of high burden VZV in patient’s CSF. Lastly, CNS infections may be simultaneously caused by multiple pathogens in HIV-infected patients as they are immunocompromised [15]. Those CNS mixed infections may be simultaneously identified in a single run with mNGS technology.

It should be noted that mNGS also has some limitations such as human genome contamination and possibly environmental microbial contamination [9]. The vast majority of reads in mNGS are derived from human host. This would impede the overall analytical sensitivity of mNGS for pathogen detection. Host depletion methods or targeted sequencing may help to partially mitigate this disadvantage [9]. As mNGS could not, by itself, define whether the detected microbe is the causative pathogen or environmental microorganism, a multidisciplinary discussion by clinicians, microbiologists as well as the lab technicians is required to interpret the result. Before this new approach could be considered as a front-line diagnostic test in HIV-infected patients with CNS infections, the cost and availability should be furthered improved.

In conclusions, in the current study, a fulminant CNS VZV infection without typical rash in an HIV-infected patient was unexpectedly diagnosed by mNGS. This study suggests that mNGS may be a useful diagnostic tool for CNS VZV infection. More importantly, as mNGS does not require a prior knowledge of causative pathogens and could identify all pathogens directly from CSF sample in a single run, it has the promise of strengthening our ability to diagnose CNS infections in HIV-infected patients.

## Data Availability

The classification reference databases were downloaded from National Center Biotechnology Information (ftp://ftp.ncbi.nlm.nih.gov/genomes/). The VZV sequencing data from our case have been uploaded to NCBI with Submission ID SUB6934634, BioProject ID PRJNA605297. The other datasets used and/or analyzed during the current study are available from the corresponding author on reasonable request.

## Abbreviations

CMV: Cytomegalovirus
CNS: Central nervous system
CSF: Cerebrospinal fluid
HBV: Hepatitis B virus
HIV: Human immunodeficiency virus
mNGS: Metagenomic next-generation sequencing
PCR: Polymerase chain reaction
VZV: Varicella-zoster virus
